# The Spiritual Needs Questionnaire in Research and Clinical Application: a Summary of Findings

**DOI:** 10.1007/s10943-021-01421-4

**Published:** 2021-09-07

**Authors:** Arndt Büssing

**Affiliations:** Professorship Quality of Life, Spirituality and Coping, Witten/Herdecke University, Gerhard-Kienle-Weg 4, 59313 Herdecke, Germany

**Keywords:** Spiritual needs, Assessment, Questionnaire, Spiritual care, Quality of life

## Abstract

To provide spiritual care, an assessment and documentation of patients´ spiritual struggles and/or their spiritual needs is required to initiate appropriate support planning processes. For that purpose, the *Spiritual Needs Questionnaire* (SpNQ) was developed in 2009 as an easy to apply standardized measure. The tool has so far been translated into numerous languages and is widely used as a valid and reliable instrument to assess a wide range of spiritual needs of patients with chronic diseases, elderly, adolescents, and healthy persons. Its four main factors address *Religious needs*, *Existential needs*, *Inner Peace needs*, and *Giving/Generativity* needs. Here, the main findings are summarized and discussed.

## Background

Spiritual care implies that one tries to address patients´ spiritual struggles, fears and worries, to listen to their spiritual needs, and to support their underlying spirituality, whatever this may mean to them. Even non-religious persons may have specific religious and non-religious spiritual needs (Büssing et al., 2013a). As spirituality is a complex and multidimensional construct with different layers and meanings (Büssing, 2019; Emmons, 2000; Zwingmann et al., 2011), there are different opinions of what spiritually concretely means to address this topic in health care and psychology (Ross, 2006; Nolan, 2021; Frick, 2017; Ai et al., 2021), and by whom it should be addressed (i.e., pastoral workers, nurses, physicians, psychotherapists, social workers) (Handzo & Koenig, 2004). Of course, certified pastoral workers are trained to respond to spiritual struggles and to listen and respond to patients´ spiritual needs (Handzo & Koenig, 2004; Puchalski et al., 2009). However, several patients (particularly in less religious societies) are reluctant to talk about their concerns with church-based pastoral/spiritual care workers, particularly when they reject the church as an institution, and thus nurses, psychologists and physicians might be in charge and have to address this topic. Findings from Balboni et al. (2007) indicated that a majority of tumor patients did not or minimally believed their spiritual needs were supported by the medical system, and about half of them did not feel supported by a religious community which might be considered as being ‘responsible’ to care for these issues. This leaves a large proportion of patients alone with their spiritual needs, as it is not too surprising that stressed medical doctors and nurses may not have time (or competence) to respond to their patients´ spiritual needs. However, even in rather secular Germany, most tumor patients stated that their medical doctors should show interest in their spiritual orientation (Frick et al., 2006), and this implies that medical professionals should be open to this topic and to proactively invite patients that value this aspect of their life which is seen as important in the process of recovery or coping and could thus be addressed. Among US American outpatients from family practices, 30% wanted their physicians to “address religious issues with them” (Maugans & Wadland, 1991). Qualitative findings from Grant et al. (2004) indicated that patients with life–threatening diseases “were best able to engage their personal resources to meet their needs when affirmed and valued by health professionals”. In fact, there might be an implicit demand that health care professionals should recognize and address these specific needs, as for example 23% of German patients with chronic pain diseases intended to talk with a pastor/pastoral worker about their spiritual needs, while 20% had no one to talks with about these issues, and 37% wanted to talk about their spiritual needs with their medical doctor (Büssing et al., 2009). Yet, medical doctors have arguments on their side to not address these issues (i.e., professional neutrality, lack of time, lack of expertise, or even discomfort; Curlin et al., 2006; Lee & Baumann, 2013). An argument against these concerns is that spiritual care primarily means to *listen* to what is needed instead of *doing* or *knowing* something. Listening means showing interest in patients´ concerns, their fears, worries and hopes, and thus trying to find ways to support their psychosocial, existential and spiritual needs (Büssing, 2021a). This process of listening requires also an assessment of what is important to them (Puchalski, 2021), and then adequate support by an interprofessional spiritual care team (Büssing, 2021b).

## Development of the Spiritual Needs Questionnaire

**Fig. 1:**
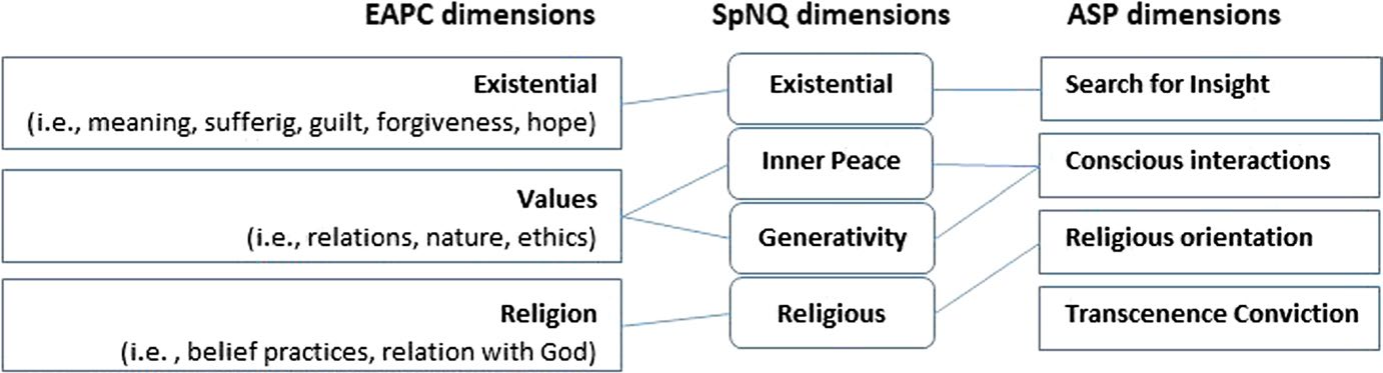
Topics of the SpNQ and their relation to dimensions defined by the EAPC and identified as aspects of spirituality (ASP) in a general population. As transcendence convictions were of minor relevance in different samples, this topic was not included in the SpNQ

To address patients´ spiritual needs in a standardized way that is easy to apply by health care professionals, and will not require specific (pastoral) competencies, the *Spiritual Needs Questionnaire* (SpNQ) was developed in 2009 (Büssing et al., 2010). It was developed to cover a wide spectrum of spiritual needs that may be relevant also for non-religious persons, who often feel offended by religious issues and thus will not even consider to talk about their concerns when they assume it is all about ‘church’ and proselytizing. Further, some (often non-religious) health care professionals have objections against the topic of ‘religion’ (Lee & Baumann, 2013), and to convince them that the topic of spirituality is more than explicit religious concerns, the instrument covers both, secular aspects and religious issues. This intermix of topics has raised the instruments´ acceptance by health care professionals. A further argument for the heterogeneity of different needs addressed by the instrument is that the construct Spirituality is in fact multidimensional (Zwingmann et al., 2011), and that the different dimensions are differentially relevant to persons from secular societies (Büssing et al., 2007). As there are different positions, in order to determine which of the different aspects of the multidimensional construct Spirituality should be seen as ‘truly spiritual’ and which not, it was necessary for the content of the term ‘Spirituality’ to be assessed in a general population of religious persons, non-religious but spiritual persons, and atheists/non-believers (Büssing et al., 2007). The identified motifs can be condensed to four main topics: Religious Orientation (i.e., faith, praying, trust in God), Search for Insight/Wisdom (which is an existential issue, i.e., insight and truth, beauty and goodness, search for existential answers), Conscious Interactions (i.e., conscious interactions with others, self, and environment, compassion and generosity), and Transcendence conviction (i.e., belief in the existence of higher beings, rebirth of man/soul) (Büssing et al.,). These aspects of spirituality inspired the four core dimensions of the Spiritual Needs Questionnaire (i.e., Connection, Peace, Meaning/Purpose and Transcendence) that relate to the categories of social, emotional, existential and religious needs (Büssing & Koenig, 2010) (Fig. 1).

Also, the European Association for Palliative Care (EAPC) has underlined that the “spiritual field is multidimensional” and that it covers “Existential challenges (e.g., questions concerning identity, meaning, suffering and death, guilt and shame, reconciliation and forgiveness, freedom and responsibility, hope and despair, love and joy). Value based considerations and attitudes (what is most important for each person, such as relationships with oneself, family, friends, work, nature, art and culture, ethics and morals, and life itself); Religious considerations and foundations (faith, beliefs and practices, the relationship with God or the ultimate)” (EAPC, no date). These main dimensions are also addressed by the SpNQ (Fig. 1).

The instrument is primarily intended to assess the intensity of specific needs (also in terms of a documentation system) and as an opportunity to start communication about patients´ specific needs, while it is also an instrument to facilitate research on this topic and to differentiate the respective needs in different groups of patients, stages of disease, cultures and religions. Therefore, the SpNQ can be used as a resource oriented ‘diagnostic’ instrument with 27–30 items (and three free text fields to encourage a reflection about additional specific needs of importance) which also includes ‘non-spiritual’ topics to facilitate its application, or as a 20-item research instrument (Table 1) with four main dimensions. The items were introduced by the phrase “During the last time, did you have had the needs …” or specifically: “…during the last 2 weeks…”. The intensity of the respective needs is rated on a 4-point scale (0—*not at all*; 1—*somewhat*; 2—*strong*; 3—*very strong*). The specifically religious needs were placed in the second half of the questionnaire, as this raised the acceptance of non-religious persons to respond to items, including the religious needs items.

**Table 1:** Items of the SpNQ and their usage in the three different versions

During the last time/during the last 2 weeks, did you have had the needs …		SpNQ-20	SpNQ-Ad	SpNQ Screener
N2	To talk with others about your fears and worries?	IPN	APC	IPN
N3	That someone of your religious community (i.e., pastor) cares for you or come to see you?	*	–	–
N4	To reflect back on your life?	*	–	–
N5	To dissolve/clarify open aspects of your life?	*	–	–
N6	To plunge into beauty of nature?	IPN	IPN	–
N7	To dwell at a place of quietness and peace?	IPN	IPN	IPN
N8	To find inner peace?	IPN	IPN	IPN
N10	To find meaning in illness and/or suffering?	ExN	EGN	–
N11	To talk with someone about the question of meaning in life?	ExN	EGN	ExN
N12	To talk with someone about the possibility of life after death?	ExN	–	–
N13	To turn to someone in a loving attitude?	*	APC	–
N14	To give away something from yourself?	GGN	–	–
N15	To give solace to someone?	GGN	–	–
N16	To forgive someone from a distinct period of your life?	ExN	–	ExN
N17	To be forgiven?	ExN	–	ExN
N18	To pray with someone?	RN	RPN	–
N19	That someone prays for you?	RN	RPN	–
N20	To pray for yourself?	RN	RPN	RN
N21	To participate at a religious ceremony (i.e., Sunday service)?	RN	–	RN
N22	To read religious/spiritual books?	RN	–	–
N23	To turn to a higher presence (i.e., God, Allah, Angels, Saints)?	RN	RPN	RN
N25	To feel connected with family?	*	–	–
N26	To pass own life experiences to others?	GGN	–	–
N27	To be assured that your life was meaningful and of value?	GGN	EGN	–
N28	To be re-involved by your family in their life concerns?	*	–	–
N29	To be invited by friends?	*	–	–
N30	To receive more support from your family?	*	–	–
N38	That there is someone there for you who is always at your side?	–	APC	–
N43	That your situation is improving for the better?	–	APC	–
N44	To have someone to assure you how to proceed (in a positive way)?	–	APC	–

SpNQ © Arndt Büssing, www.spiritualneeds.net permission required to copy or publish

^*^Items are not used to calculate the SpNQ-20 scores, but are nevertheless clinically relevant and are used as additional information

Abbreviations: The SpNQ-20 differentiates *RN* religious needs, *ExN* existential needs, *IPN* inner peace needs, *GGN* giving/generativity needs. The SpNQ-Ad differentiates *RPN* religious/praying needs, *EGN* existential/giving needs, *APC* attention/positive confirmation, and *IPN* inner peace needs

## Factorial Structure of the Spiritual Needs Questionnaire

The 20-item research version (SpNQ-20) differentiates four main factors: *Religious* *needs*, *Existential* *needs*, *Inner Peace* *needs*, and *Giving/Generativity* *needs* (Table 1). These were finally verified in large samples of patients with chronic diseases, elderly, and healthy (stressed) persons (Büssing et al., 2018a). Internal consistencies of the four factors range, depending on the samples, from Cronbach´s alpha = 0.71 to 0.87. Among the additional items, three address relational support (Table 1), which is an important topic not only for psychologists and social workers, but is not specifically a spiritual topic. As these had a less satisfying internal consistency, they are not officially used as scale.

There are different translations of the SpNQ available (Riklikiene et al., 2021), and the respective researchers have validated a varying number of items, which makes it difficult to compare findings. Therefore, it is recommended to also report the SpNQ-20 Mean Score (Cronbach´s alpha = 0.88) which was 1.01 ± 0.63 (median = 0.90; 25% percentile = 0.52, 75% percentile = 1.42) in the validation sample (Büssing, 2021d).

Meanwhile, there are also other approaches to assess a person´s spiritual needs published, such as the 23-item *Spiritual Needs Assessment for Patients* (SNAP) (Sharma et al., 2012), the 28-item *Spiritual Needs Questionnaire for Palliative Care* (SNQPC) (Vilalta et al., 2014), the 12-item *Psychosocial and Spiritual Needs Evaluation Scale* (ENP-E) (Mateo-Ortega et al., 2018), and previous assessments such as the 17-item *Spiritual Needs Inventory* (SNI) (Herman, 2006), the 29-item *Spiritual Needs Survey* (SNS) (Galek et al., 2005) or the Korean language *Spiritual Needs Scale* (SNS) (Yong et al., 2008), which are predominantly intended to be used at patients´ end of life. A benefit of the SpNQ is that it is not restricted to end-of-life issues, but also relevant for more early and less severe stages of disease, and applicable in adolescents and in healthy persons in difficult life situations (Büssing et al., 2021a, 2021b; Büssing, 2021d, 2021e). The SpNQ instrument is available in different translations and is used as a research tool in international studies [overview in Büssing (2021c)], and was highlighted as a relevant spiritual needs assessment in literature/scoping reviews (Seddig et al., 2016; Nissen et al., 2020).

## Application of the Instrument in Different Countries

So far, the following language versions are published: German (Büssing et al.,), Polish (Büssing et al., 2015), Danish (Stripp et al., submitted), Lithuanian (Riklikienė et al., 2019), Croatian (Glavas et al., 2017), Greece (Fradelos et al., 2020), Portuguese (de Abreu et al., 2021; Riklikiene et al., 2021), Brazilian Portuguese (Oliveira da Silva et al., 2020; Valente et al., 2018), Chinese (Büssing et al., 2013b; Zhao et al., 2019), Indonesian (Himawan et al., 2019; Nuraeni et al., 2015; Sastra et al., 2021), Iran/Farsi (Hatamipour et al., 2018; Moeini et al., 2018), Pakistanian (Kashif & Kanwal, 2018). Other translations are in use but not yet published (i.e., Italian, French, Spanish, Japanese and Korean).

Depending on the cultural and religious context, the sample size and population, and the number of items used by the respective researchers (even when it is recommended to use the 20 established items, because adding or deleting important items may change the factorial structure), the factorial structure of the different versions may vary to some extend (Riklikiene et al., 2021). For example, items addressing family concerns are not necessarily a spiritual issue, but may nevertheless be of relevance for the patients, and adding these items to the item pool will change the factorial structure of the respective language version. Further, some assumingly existential needs items may be both, an existential need and an inner peace need, depending on a person´s age, dynamics of the disease, or their expectations, and thus such items may switch from one scale to the other (Büssing, 2021d).

## Additional Versions of the SpNQ

In some specific contexts, it may be important to adjust the item pool to the underlying intention. The adolescents´ version of the SpNQ (SpNQ-Ad) avoids conflicting (‘end of life’) items and adds three additional items (“that your situation is improving for the better”; “to have someone to assure you how to proceed (in a positive way)”; “that there is someone there for you who is always at your side”) (Büssing, 2021e). This 15-item SpNQ-Ad differentiates *Religious/Praying needs*, *Existential/Giving needs*, *Needs for Attention/Positive Confirmation*, and *Inner Peace needs* with good internal consistencies (Cronbach´s alpha ranging from 0.72 to 0.89) (Table 1). The new 4-item scale *Needs for Attention/Positive Confirmation* (Cronbach´s alpha = 0.82) addresses adolescents´ hope and positive expectations (the three new items and the ‘inner peace’ item N2 “to talk with someone about your fears and worries”). The SpNQ-Ad subscales correlate with adolescents´ life satisfaction and symptom burden (Büssing et al., 2021a; Büssing, 2021e).

An important argument of palliative care providers is that several of their patients are physically and mentally too week, so there was a need for a short ‘Screener’ version of the SpNQ. This *SpNQ Screener* uses 10 items only, is not addressing the topic of Generativity (Table 1), and has nevertheless a good internal consistency (Cronbach´s alpha = 0.86) (Büssing, 2021f). Although the primary topics are addressed (*Inner Peace* needs with 4 items, *Existential* needs with 3 items, and *Religious* needs with 3 items), it is recommended to use a sum-score ranging from 0 to 30 (mean score in the validation sample: 11.5 ± 7.4) which makes it more easy to handle. Interestingly, the sum scores of palliatively treated patients differed only weakly from that of patients with more early stages (who had marginally higher needs) (Büssing, 2021f). This underlines that spiritual needs should not only be addressed and supported in the palliative situation, but very early (after diagnosis) in order not to leave patients alone in their difficult situation and to be available as the first point of contact. This shortened *Screener* version is not intended to replace the 20-item SpNQ, but as an optional version to start communication with patients in far advanced stages of their disease (i.e., in hospice).

## Intensity of Spiritual Needs

The SpNQ was applied in different groups of persons: Patients with cancer, chronic pain diseases, kidney diseases, psychiatric conditions, dementia, HIV, cystic fibrosis, persons with autism, adolescents with chronic diseases, mothers with sick newborns, healthy but stressed soldiers, war victims and refugees, patients in emergency rooms, patients´ relatives, and elderly in retirement homes (Büssing, 2021c). In most cases, *Inner Peace needs* and *Giving/Generativity needs* scored highest in the different samples, while *Religious needs* and *Existential needs* scored lower (Büssing, 2021c). However, in Catholics from Poland, *Religious needs* scored much higher as compared to persons from more secular societies (Büssing et al., 2015). In Muslims from Iran, *Existential needs* scored highest (Rassouli et al., 2021), while in Muslims from Croatia and Bosnia–Herzegovina, both their *Religious needs* and *Existential needs* scored high (Glavas & Baumann, 2021). Therefore, the religious and cultural context should be considered when the findings are interpreted.

Women usually express more intense spiritual needs than men, and persons with higher age (Büssing, 2021d). As one may expect, non-religious persons score low on *Religious needs*, marginally lower on *Existential needs*, but did not significantly differ from their religious counterparts with respect to their *Inner Peace needs* and *Giving/Generativity needs* (Büssing, 2021d). Also, those without religious affiliations have specific spiritual needs, and thus is it worth to assess their spiritual needs too; in fact, there is a quite large fraction within this assumingly non-religious group of persons that may have even specific religious needs (Büssing, 2021d).

## Spiritual Needs and Their Associations with Indicators of Spirituality

Are all these needs addressed by the SpNQ related to spiritual issues? It was found that in patients from Catholic Poland, *Religious needs* were strongly related to Religious Attitudes, spiritual Search (for spirituality as a resource), religious Trust, positive emotions toward God, and moderately to Ethical Sensitivity and (spiritual) Harmony, while *Giving/Generativity needs* were moderately related to religious Trust and Ethical sensitivity, and weakly to Religious Attitudes, Search, and to positive emotions toward God (Büssing et al., 2015). In contrast, *Existential needs* were weakly related to Search and Trust, and marginally only to positive emotions toward God, while *Inner Peace needs* were weakly only related to Search. Thus, in patients from Catholic Poland, the secular dimensions *Existential* needs and *Inner Peace* needs were understood as rather non-religious spiritual needs, which were nevertheless of relevance to them.

In patients with fibromyalgia from secular Germany, *Religious needs* were strongly related to religious Trust and moderately to spiritual Search; *Existential needs* were moderately related to both dimensions of spirituality, while *Inner Peace needs* and *Giving/Generativity needs* were weakly only, or not at all, related to spiritual Search or religious Trust (Offenbaecher et al., 2013). A similar pattern was found in patients with various chronic pain conditions from Germany (Büssing et al., 2013a). Thus, in patients from secular Germany, also *Existential* needs are seen as a rather spiritual issue, but not *Inner Peace* needs. The interpretations whether a specific need may be a spiritual/existential or a psychosocial issue depends on the religious background. The topic of forgiveness for example may be a religious issue for Catholics, while for non-religious people, it could be an existential issue as it may indicate the needs to clarify open aspects in life (Büssing, 2021d; Riklikiene et al., 2021). Further, one may argue that needs for inner peace are a psychosocial issue. This might be right in principal, but the scale addressed also the intention to experience the beauty of nature and to dwell at a place of quietness and peace (which is mostly found in nature), and thereby to find inner peace. Interestingly, pausing in wondering awe is mostly triggered by touching experiences in nature, also by non-religious persons, and thus the respective inner peace needs related to nature, quietness and peace may be an implicit search for ‘sacred moments’—and are thus a spiritual issue, too (Büssing et al., 2018b; Keltner & Haidt, 2003).

## Spiritual Needs and Their Associations with Quality of Life indicators

Depending on the course of disease, life situation, cultural context and age, the correlative associations between spiritual needs and health indicators are heterogeneous.

In elderly from three different European countries living in retirement homes, their spiritual needs were weakly to moderately positively related to negative mood states (i.e., hopelessness, emotional tiredness, grief), to loneliness and to daily life affections, and negatively to their self-care abilities and multidimensional life satisfaction (Büssing et al., 2021e). The triggers of their spiritual needs are thus those aspects that are lacking in their life, i.e., independence, vitality, wellbeing and health.

In persons with chronic pain diseases, particularly *Inner Peace needs* and *Existential needs* were moderately positively related to indicators of low mental health (i.e., anxiety, depressive symptoms, catastrophizing) and to their intention to escape from their life situation/illness, while *Religious needs* were not significantly related to these indicators (Offenbaecher et al., 2013). Further, none of the spiritual needs was significantly related to patients´ physical health related quality of life (Offenbaecher et al., 2013).

In patients with cancer, anxiety predicted their *Existential needs* and *Inner Peace needs*, while their *Religious needs* were not relevantly related to negative mood states (Höcker et al., 2014). In cancer patients from Lithuania, only their *Religious* *needs* were at least marginally negatively related to their life satisfaction, but none of the other needs (Riklikienė et al., 2020). Further, cancer patients with high pain perception and those who are dependent on the help of others had significantly higher spiritual needs than their less burdened counterparts (Riklikienė et al., 2020).

In patients with cystic fibrosis, who were replying to the SpNQ-Ad questionnnaire, particularly their *Attention/Positive Affirmation needs* and *Existential/Giving needs* were moderately negatively related to their life satisfaction, while their *Inner Peace needs* were weakly inversely related, and *Religious/Praying needs* not at all (Große-Onnebrink & Büssing, 2021). Among the clinical parameters, lung function scores of patients with cystic fibrosis were weakly related to *Inner Peace needs* (Große-Onnebrink & Büssing, 2021).

In patients with psychiatric diseases, their spiritual needs were minimally only related to their life satisfaction or depressive symptoms, while *Inner Peace needs* were weakly related to distress (Baumann et al., 2021). These patients were in treatment and thus their symptoms were mitigated; therefore, it is not that surprising that their spiritual needs were less related to their quality of life. In persons with autism disorders, life satisfaction was weakly related to *Existential needs*, while finding meaning in life was moderately related to *Religious needs*, and weakly to their *Existential needs* (Cwik, 2021).

The spiritual needs of refugees coming from different countries, which scored high on all spiritual needs dimensions, were marginally only related to their life satisfaction, which scored differentially depending from their home country and thus cultural context (Maier & Surzykiewicz, 2021). In that sample, the home country was the moderator of the link between spiritual needs and life satisfaction.

In healthy soldiers, stress perception and posttraumatic stress disorder (PTSD) symptoms were moderately positively related to their *Inner Peace needs* and *Existential needs*, while their multidimensional life satisfaction was weakly to moderately negatively related to *Existential needs* and *Inner Peace needs* (Büssing & Recchia, 2016; Büssing, 2021d). In healthy but stressed young mothers with sick children, *Inner Peace needs* were moderately associated to their perceived stress and weakly with grief, while none of the other needs were significantly related to stress perception or mood states, and none of the spiritual needs was related to their life satisfaction (Büssing et al., 2017).

However, the way patients see and interpret their disease and life situation is crucial for their coping processes. It was found that particularly positive interpretations of illness such as Challenge and Value (to grow on) were moderately positively related to *Religious needs* and *Existential needs*, while guilt-associated negative interpretations were not at all or marginally only associated with spiritual needs (Büssing et al., 2013a; Büssing, 2021d). This would indicate that patients still see their life as worth it, as a challenge and something of value to fight for and to grow on—and that they are in search for resources to rely on and thereby able coping with their life situation (Büssing, 2021d).

## Spiritual Needs and Spiritual Wellbeing

It is important to underline that spiritual needs are not necessarily a reaction to low spiritual wellbeing. In palliatively treated patients from Germany, their *Existential* *needs* and *Inner Peace* *needs* were negatively (and this would be plausible from a theoretical point of view), but marginally only related to spiritual wellbeing as measured with the FACIT-Sp12 (Bredle et al., 2011), while the faith component of spiritual wellbeing was positively (which would be contra-intuitive) and strongly related to their *Religious* *needs* (Büssing et al., 2020). Also in patients with chronic pain conditions, this distinct correlation pattern was observed (Büssing et al., 2013a). In German mothers with sick newborns or premature children, *Religious* *needs* were strongly positively and *Giving/Generativity* *needs *weakly positively associated with the Faith dimension of spiritual wellbeing (as measured with the FACIT-Sp12, too), while their *Existential* *needs* were not relevantly related to the Meaning or Peace component of spiritual wellbeing, and *Inner Peace* *needs* marginally negatively only related with the Peace component (Büssing et al., 2017). In contrast, in mothers of sick children from Catholic Lithuania, their spiritual needs were moderately to strongly positively related to the transcendental and the communal aspects of spiritual wellbeing (Büssing & Riklikine, 2021), and this would again be contra-intuitively. In that study, the SHALOM questionnaire was used to asses spiritual wellbeing (Fisher, 2021). While the negative associations between spiritual wellbeing and spiritual needs are sound from a theoretical point of view (because that what is lacking will trigger the respective needs), the positive association between spiritual wellbeing and *Religious* needs requires an explanation. Person who can utilize their faith as a resource, particularly in secular societies, may be generally well with their religious life and their faith, and thus they would express religious needs because they have this resource to rely on, and thus the correlation is positive. A further argument could be that “the construct of ‘spiritual wellbeing’ is related to psychological wellbeing in dealing with issues related to meaning and purpose in life, while having unmet ‘spiritual needs’ is a broader concept that indicates a general longing to fill unmet psychological, social, and personal needs related to the transcendent or the immanent” (Büssing & Koenig, 2021).

## Application of the Instrument

Handling the 1-page instrument to the patients leaves it up to them to reflect on their needs and to decide whether they return the filled instrument to the health care team or not, thereby indicating a willingness to talk about their spiritual needs. With this knowledge, the multiprofessional health care/spiritual care team (who all contribute with their specific profession-related expertise and individual competence) is able to plan the subsequent processes in response to the stated spiritual needs, to develop a support plan and consent it with the patients as described (Büssing, 2021b; Wordsworth, 2021). Topics of such a support plan are discussed elsewhere (Büssing, 2021b). The application of the SpNQ in the care plan of parish nurses was describe by Wordsworth (2021), and comments of the applicants were added that described it as a useful tool for their work.

It is so far unclear whether a person´s longing in terms of unmet needs will in fact decline (or can be ‘fulfilled’ at all) when the respective needs are addressed. Some needs might be desires that cannot be fulfilled at all, while others can be principally addressed and thereby may lose their intensity. In palliative care patients, it was shown that during their stay at the Palliative Care Unit, neither their spiritual needs nor their spiritual wellbeing changed significantly within the short time span of two or three weeks, while, however, patients were nevertheless highly satisfied with the supported of the hospital team (Büssing et al., 2020).

Experiences with the instrument at Palliative Care Units indicate that the topic of spiritual needs might be ‘very close’ to some patients and that several of them put it away until they were physically and emotionally more stable to respond; but then they noticed that it was good “to face these issues” (Büssing et al., 2021c). Jochen Rentschler reported that “there were many in-depth discussions with the nursing staff or the doctors about points that had particularly touched and concerned the patients”, and that conventional spiritual anamnesis was not that profound as the talks with the patients were in response to the SpNQ (Büssing et al., 2021c). This was the intention, to apply the instrument as an opportunity to communicate about those things that are also important to the patients but often ignored during the routine in hospitals. Staff of the palliative care centers noticed that using the instrument “noticeably increased both the quantity and the quality of the spiritual care provision” (Büssing et al., 2021c). A chaplain from Hamburg commented that the topics addressed by the instrument acquired “a depth and emotionality” that can hardly be dealt by the nursing staff and can hardly be adequately discussed without conversation training. This might be true and is a further argument for specific spiritual care training (O´Brien et al., 2019; Appleby et al., 2019; Alt-Epping et al., 2021). Staff´s spiritual care competences could be assessed with the *Spiritual Care Competence Questionnaire* (SCCQ) to identify training needs and to evaluate spiritual care training programs (Frick et al., 2019; Pastrana et al., 2021).

## Conclusions

The *Spiritual Needs Questionnaire* (SpNQ) was developed as an easy to apply standardized measure to be used in both, secular and religious societies. It is so far translated in different languages and widely used as a valid and reliable instrument to assess the spiritual needs of patients with chronic diseases, elderly, adolescents and healthy persons. Its four main factors address *Religious* *needs*, *Existential* *needs*, *Inner Peace* *needs*, and *Giving/Generativity* *needs*. The instrument is applicable also to non-religious persons who also have spiritual needs, particularly needs for inner peace and generativity (Büssing et al., 2013a), while it is of relevance also for religious persons who score higher on explicit religious needs (Büssing et al., 2013a, 2015; Himawan et al., 2019; Rassouli et al., 2021). Depending on the sample of affected persons, their spiritual needs may be triggered by reduced mental health, negative mood states and low life satisfaction, and by their interpretation of illness; yet this is not a prerequisite as also other factors may be relevant, such as activity of disease, available resources and support, personality and spiritual ‘resonance’ or interest (Büssing & Koenig, 2021). The standardized SpNQ-20 Sum Score can be used to compare data from different religious and cultural contexts, when the respective culturally adapted versions may differ in terms of item numbers and factorial structure. With this instrument, even health care professionals who are not yet trained in spiritual care can easily assess their patients´ unmet spiritual needs and can start communication about those concerns that are also important to patients during times of illness. Addressing these needs nevertheless requires multiprofessional teams with specific spiritual care training to avoid individual excessive demands.
